# The Diversity of Bacteria Associated with the Invasive Gall Wasp *Dryocosmus kuriphilus*, Its Galls and a Specialist Parasitoid on Chestnuts

**DOI:** 10.3390/insects13010086

**Published:** 2022-01-13

**Authors:** Xiaohui Yang, Yu Hui, Daohong Zhu, Yang Zeng, Lvquan Zhao, Xuemei Yang, Yumei Wang

**Affiliations:** 1 College of Life Science, Hunan Normal University, Changsha 410081, China; yuhui@hunnu.edu.cn (Y.H.); xuemeiyang@hunnu.edu.cn (X.Y.); wym@hunnu.edu.cn (Y.W.); 2 Laboratory of Insect Behavior and Evolutionary Ecology, Central South University of Forestry and Technology, Changsha 410004, China; dhzhu@csuft.edu.cn (D.Z.); t20162281@csuft.edu.cn (Y.Z.); 3 Co-Innovation Center for Sustainable Forestry in Southern China, College of Forestry, Nanjing Forestry University, Nanjing 210037, China; zhaolvquan@njfu.edu.cn

**Keywords:** *Dryocosmus kuriphilus*, *Torymus sinensis*, diversity, bacterial community, galls, high-throughput sequencing, *Castanea mollissima*

## Abstract

**Simple Summary:**

The insect *Dryocosmus kuriphilus* induces galls on chestnut trees. *Torymus sinensis* is a host-specific parasitoid of *D. kuriphilus* and phenologically synchronizes with *D. kuriphilus*. The aim of this research is to investigate the bacterial communities and predominant bacteria of *D. kuriphilus*, *T. sinensis*, *D. kuriphilus* galls and the galled twigs of *Castanea mollissima*. We provide the first evidence that *D. kuriphilus* shares most bacterial species with *T. sinensis*, *D. kuriphilus* galls and galled twigs. The predominant bacteria of *D. kuriphilus* are *Serratia* sp. and *Pseudomonas* sp. Many species of the *Serratia* and *Pseudomonas* genera are plant pathogenic bacteria, and we suggest that *D. kuriphilus* may be a potential vector of plant pathogens. Furthermore, a total of 111 bacteria are common to *D. kuriphilus* adults, *T. sinensis*, *D. kuriphilus* galls and galled twigs, and we suggest that the bacteria may transmit horizontally among *D. kuriphilus*, *T. sinensis*, *D. kuriphilus* galls and galled twigs on the basis of their ecological associations.

**Abstract:**

*Dryocosmus kuriphilus* (Hymenoptera: Cynipidae) induces galls on chestnut trees, which results in massive yield losses worldwide. *Torymus sinensis* (Hymenoptera: Torymidae) is a host-specific parasitoid that phenologically synchronizes with *D. kuriphilus*. Bacteria play important roles in the life cycle of galling insects. The aim of this research is to investigate the bacterial communities and predominant bacteria of *D. kuriphilus*, *T. sinensis*, *D. kuriphilus* galls and the galled twigs of *Castanea mollissima*. We sequenced the V5–V7 region of the bacterial 16S ribosomal RNA in *D. kuriphilus*, *T. sinensis*, *D. kuriphilus* galls and galled twigs using high-throughput sequencing for the first time. We provide the first evidence that *D. kuriphilus* shares most bacterial species with *T. sinensis*, *D. kuriphilus* galls and galled twigs. The predominant bacteria of *D. kuriphilus* are *Serratia* sp. and *Pseudomonas* sp. Furthermore, the bacterial community structures of *D. kuriphilus* and *T. sinensis* clearly differ from those of the other groups. Many species of the *Serratia* and *Pseudomonas* genera are plant pathogenic bacteria, and we suggest that *D. kuriphilus* may be a potential vector of plant pathogens. Furthermore, a total of 111 bacteria are common to *D. kuriphilus* adults, *T. sinensis*, *D. kuriphilus* galls and galled twigs, and we suggest that the bacteria may transmit horizontally among *D. kuriphilus*, *T. sinensis*, *D. kuriphilus* galls and galled twigs on the basis of their ecological associations.

## 1. Introduction

Plant galls are outgrowths of plant tissues induced by a wide variety of organisms, including protists, nematodes, mites, fungi, bacteria and insects [1]. Galling insects manipulate the development of host plants and induce galls on different organs of the host plants [2]. Galling insects are a highly sophisticated herbivore group, including gall wasps, gall midges, gall aphids, gall moths, thrips and psyllids [3].

The gall wasp *Dryocosmus kuriphilus* (Hymenoptera: Cynipidae) is native to China and has rapidly spread throughout Asia, Europe and North America [4,5]. *D. kuriphilus* is a univoltine and parthenogenetic species that lays eggs in the buds of *Castanea* spp. during the summer, and their larvae overwinter inside the buds and induce galls on the host plants during the following spring [6]. Galls are the sole food sources for *D. kuriphilus* [7,8]. *Torymus sinensis* (Hymenoptera: Torymidae) is a univoltine and host-specific parasitoid that phenologically synchronizes with *D. kuriphilus* [9]. During April and May, the female adults of *T. sinensis* lay eggs in newly formed galls or on the bodies of *D. kuriphilus* larvae [10,11]. After hatching, the larvae of *T. sinensis* feed ectoparasitically on the mature larvae of *D. kuriphilus*. In the winter, the female adults of *T. sinensis* overwinter inside *D. kuriphilus* galls [11]. In the following spring, the *T. sinensis* adults emerge from the overwintering withered galls that were induced by *D. kuriphilus* the previous year [12,13]. Thus, the ecological interactions among *D. kuriphilus*, *T. sinensis*, *D. kuriphilus* galls and galled twigs include oviposition, gall formation, parasitism and feeding [14].

Bacteria play important roles in the life cycle of galling insects [15]. For example, *Microbacterium* sp. of *Leptocybe invasa* can resist the secondary metabolites of plants and expand the range of host feeding, which is beneficial for the colonization of *L. invasa* [16,17]. *Buchnera aphidicola* synthesizes essential amino acids for gall aphids and facilitates the stress tolerance and reproduction of the host [18,19]. Furthermore, *Wolbachia* may cause the parthenogenesis of some galling insects [20]. However, previous studies have indicated that thelytokous parthenogenesis of *D. kuriphilus* may not be associated with *Wolbachia* [21]. To date, no studies have reported the effects of bacteria on *D. kuriphilus* and *T. sinensis*.

Bacteria infect *D. kuriphilus*, *T. sinensis* and galls. Previous studies have isolated *Staphylococcus saprophyticus*, *Paenibacillus* sp., *Paenibacillus* sp. and *Pseudomonas fluorescens* from the body of *D. kuriphilus* [22]. Notably, the species of *Pseudomonas* genus are the predominant bacteria in many galling insects, including gall midges, gall aphids, gall sawflies, thrips and *L. invasa* [17,18,23,24,25,26,27]. Furthermore, *T. sinensis* are infected with the symbiotic bacteria *Wolbachia* [28]. For cynipid galls, high-throughput sequencing analyses have confirmed that the predominant endophytic bacteria of *Lithosaphonecrus arcoverticus* (Hymenoptera: Cynipidae) galls are *Allorhizobium*, *Chryseobacterium*, *Curtobacterium*, *Luteibacter*, *Pantoea* and *Stenotrophomonas* genera [29]. However, the difference between the bacterial communities of *D. kuriphilus*, *T. sinensis* and *D. kuriphilus* galls and galled twigs within the same habitat remains unclear. Furthermore, some galling insects may acquire bacteria from host plants [23,24,25,26], and it remains unclear whether *D. kuriphilus*, *T. sinensis* and *D. kuriphilus* galls acquire subsets of the bacteria present in the galled twigs

In this study, the bacteria within the body of *D. kuriphilus*, *T. sinensis*, *D. kuriphilus* galls and the galled twigs of *Castanea mollissima* were detected by high-throughput sequencing for the first time in order to explore the potential horizontal transmission of bacteria among *D. kuriphilus*, *T. sinensis*, *D. kuriphilus* galls and galled twigs. The α-diversity, community structures and predominant species of bacteria associated with *D. kuriphilus*, *T. sinensis*, *D. kuriphilus* galls and galled twigs were compared at the species level, which is the level of taxonomic classification of bacteria. We discuss horizontal transmission, differences in community structure and the predominant species of bacteria in *D. kuriphilus*, *T. sinensis*, *D. kuriphilus* galls and galled twigs on the basis of their ecological associations.

## 2. Materials and Methods

### 2.1. Collection and Pretreatment of Specimens

*D. kuriphilus* galls, the galled twigs of *C. mollissima* and overwintering withered galls were collected in Huangqiao Town (27.02° N/110.85° E), China, from April to May 2019. The *D. kuriphilus* galls grow and develop on the galled twigs (Figure S1). The overwintering withered gall refers to *D. kuriphilus* galls induced by *D. kuriphilus* in the previous year. One *D. kuriphilus* gall or overwintering withered gall can produce a single *D. kuriphilus* or *T. sinensis*, respectively. The surface of the *D. kuriphilus* gall is smooth and intact. All samples were frozen with liquid nitrogen for 30 min and transported to the laboratory on a dry ice pack, then stored at −78 °C until further processing. *D. kuriphilus* and *T. sinensis* living in galls were also frozen immediately and stored at −78 °C. The surfaces of the samples were sterilized with 75% ethanol for 2 min and 2.5% sodium hypochlorite for 5 min, then rinsed with sterile water five times. The *D. kuriphilus* galls and overwintering withered galls were removed from galled twigs, and then *D. kuriphilus* and *T. sinensis* were removed from *D. kuriphilus* galls and overwintering withered galls, respectively, with sterile scalpels. The sample size was nine for *D. kuriphilus*, *T. sinensis*, *D. kuriphilus* galls and associated galled twigs group. Each sample of *D. kuriphilus* and *T. sinensis* included 10 individuals.

### 2.2. DNA Extraction, PCR Amplification and High-Throughput Sequencing

The DNA extraction of *D. kuriphilus* female adults, *T. sinensis* female adults, *D. kuriphilus* galls and galled twigs was performed with a FastDNA^®^ Spin Kit (MP Biomedicals, Solon, OH, USA). DNA extraction blanks were used with each batch of samples to assess environmental contamination and no amplified PCR products of the DNA extraction blanks were detected.

The V5–V7 region of the bacterial 16S ribosomal RNA was amplified using nested PCR primers, with the first primer pair 799F (5′-AACMGGATTAGATACCCKG-3′)–1392R (5′-ACGGGCGGTGTGTRC-3′) and the second primer pair 799F (5′-AACMGGATTAGATACCCKG-3′)–1193R (5′-ACGTCATCCCCACCTTCC-3′). Sterile DNA-free water was used instead of template DNA as the negative control in the 16S amplicon screening process to exclude the possibility of amplicon contamination. The PCR was performed on a GeneAmp PCR System 9700 (Applied Biosystems, London, UK) in a 20 μL reaction volume: 4 μL 5×TransStart FastPfu buffer; 0.4 μL Taq polymerase; 2 μL dNTPs (2.5 mM each); 0.8 μL forward and reverse primers (5 μM); 1 μL DNA template; and 11 μL H_2_O. The cycling conditions of the first round of nested PCR were 5 min at 95 °C, followed by 27 cycles of 30 s at 95 °C, 30 s at 53 °C, 45 s at 72 °C and a final elongation step of 15 min at 72 °C. After the first round of amplification, the second round of amplification was carried out with the same cycling conditions, except that 13 cycles were performed and 1 μL of the first round PCR products was used as the template. The PCR products were separated and purified on a 2% agarose gel and AxyPrep DNA Gel Extraction Kit (Axygen Biosciences, Union City, CA, USA), then quantified with a Quantus™ Fluorometer (Promega, Madison, WI, USA).

The high-throughput paired-end sequencing was performed on the Illumina MiSeq PE300 sequencing platform (Illumina, San Diego, CA, USA) with a NEXTFLEX Rapid DNA-Seq Kit (Bioo Scientific, Austin, TX, USA) by Majorbio Bio-Pharm Technology Co. Ltd. (Shanghai, China). All raw data have been deposited in the NCBI Sequence Read Archive database under the BioProject accession number PRJNA780913.

### 2.3. Bioinformatics and Statistical Analysis

The original sequences were spliced and quality controlled with FLASH [30] and Trimmomatic [31] software according to the following criteria: sequence length >200 bp; mean quality score ≥ 20; and no ambiguous bases. Then, the high quality sequences were clustered into Operational Taxonomic Units (OTU) with a 97% similarity cutoff in Usearch software [32]. According to the Silva database, each high quality sequence was annotated at phylum, class, order, family, genus, species and OTU level using Naive Bayesian classifier [33] and the confidence threshold was 0.8. A total of 31,353 sequences were randomly selected from each sample to generate an OTU table recording the abundance and taxonomy of each OTU.

The OTU tables were imported into R version 3.6.3 (https://www.r-project.org, (accessed on 27 October 2021) for subsequent statistical analysis. We counted the number of unique and common bacteria among *D. kuriphilus*, *T. sinensis*, *D. kuriphilus* galls and galled twigs at the species level. Shannon index measures were used to evaluate the α-diversity of the bacterial communities in *D. kuriphilus*, *T. sinensis*, *D. kuriphilus* galls and galled twigs at the species level. Shapiro–Wilk and Bartlett tests showed that the distribution of the Shannon index was not normal and that the variance of the Shannon index was not homogeneous across the groups. Thus, the nonparametric Kruskal–Wallis test was used to determine whether significant overall differences existed among the Shannon index values of *D. kuriphilus*, *T. sinensis*, *D. kuriphilus* galls and galled twigs, and the Dunn test was used for multiple comparisons when the Kruskal–Wallis test result was significant. Principal coordinate analyses (PCoAs) were carried out to compare the bacterial community structures of *D. kuriphilus*, *T. sinensis*, *D. kuriphilus* galls and galled twigs. First, the overall difference in bacterial community structures was assessed with permutational multivariate analysis of variance (PERMANOVA). PERMANOVA was performed with the “adonis” function in the “vegan” package [34] in R with 1000 permutations according to the weighted UniFrac distance. Second, a PCoA was performed with the “pcoa” function in the R package “ape” according to the weighted UniFrac distance [35].

The linear discriminant analysis (LDA) effect size (LEFSE) (http://huttenhower.sph.harvard.edu/galaxy/, (accessed on 27 October 2021) was used to discover the predominant bacteria associated with *D. kuriphilus*, *T. sinensis*, *D. kuriphilus* galls and galled twigs. The Kruskal–Wallis test was used to detect bacterial taxa whose relative abundance significantly differed among *D. kuriphilus*, *T. sinensis*, *D. kuriphilus* galls and galled twigs. Then, LDA was used to calculate the effect size of each taxon; the higher the LDA score, the greater the influence of the taxa on the difference. The LDA score threshold was set to four. The predominant bacteria were those with LDA scores > 4 and a significantly higher relative abundance than that in other groups.

## 3. Results

### 3.1. Bacterial Diversity and Community Composition of D. kuriphilus, T. sinensis, D. kuriphilus Galls and Galled Twigs

**Figure 1:**
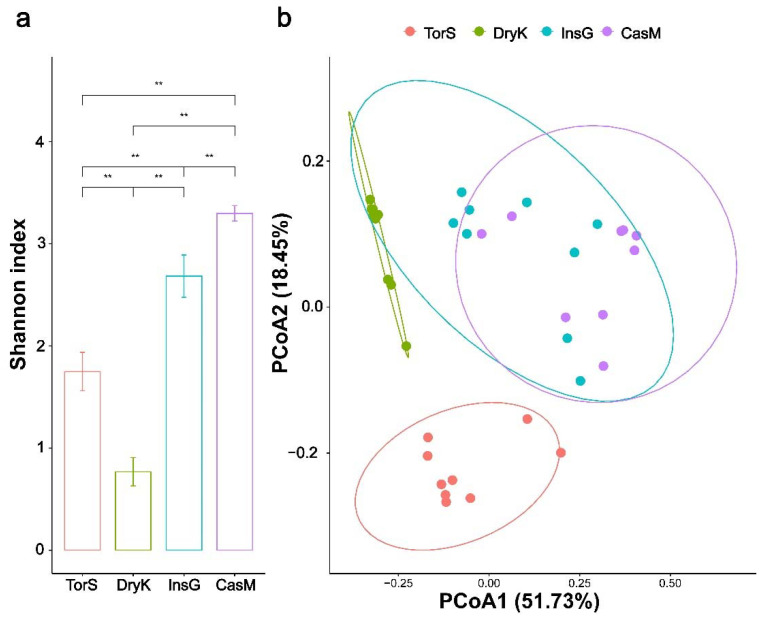
The diversity of bacterial communities in *Dryocosmus kuriphilus*, *Torymus sinensis*, *D. kuriphilus* galls and the galled twigs of *Castanea mollissima*. (**a**) The bacterial α-diversity of *D. kuriphilus*, *T. sinensis*, *D. kuriphilus* galls and galled twigs at the species level, as measured by the Shannon index. ** indicates a significant difference (*p* < 0.01). (**b**) The bacterial community structures of *D. kuriphilus*, *T. sinensis*, *D. kuriphilus* galls and the galled twigs based on the weighted UniFrac distance at the species level using Principal coordinates analysis (PCoA). The horizontal and vertical axes indicate the first and second principal coordinates (PCoA1 and PCoA2, respectively). The percentage indicates the proportion of the total variation explained by each principal coordinate. The ellipses represent the 95% confidence intervals around the centroid for *D. kuriphilus*, *T. sinensis*, *D. kuriphilus* galls and the galled twigs. DryK, TorS, InsG and CasM represent *D. kuriphilus*, *T. sinensis*, *D. kuriphilus* galls and the galled twigs of *C. mollissima*, respectively.

A total of 14 phyla, 20 classes, 63 orders, 103 families, 181 genera, 273 species and 373 OTUs were identified in the bacterial communities of *D. kuriphilus*, *T. sinensis*, *D. kuriphilus* galls and galled twigs (Table 1). Galled twigs had the most endophytic bacteria, followed by *D. kuriphilus* galls and *T. sinensis*, and *D. kuriphilus* had the fewest bacteria from the phylum to OTU level (Table 1). Similarly, the relative abundance of endophytic bacteria at the species level in galled twigs was the highest, followed by that of bacteria in *D. kuriphilus* galls, *T. sinensis* and *D. kuriphilus* (Figure 1). The changing trend of amount and relative abundance of bacteria could be associated with trophic relationships among *D. kuriphilus*, *T. sinensis*, *D. kuriphilus* galls and galled twigs. The bacterial communities of *D. kuriphilus*, *T. sinensis*, *D. kuriphilus* galls and galled twigs comprised 138, 182, 253 and 266 species, respectively (Table 1).

A significant overall difference was observed among the bacterial α-diversity (Kruskal–Wallis H test, H_3,32_ = 28.34, *p* < 0.01) and community structures (PERMANOVA, R^2^ = 0.60, *p* < 0.01) of *D. kuriphilus*, *T. sinensis*, *D. kuriphilus* galls and galled twigs (Figure 1). The α-diversity of the bacterial community in *D. kuriphilus* was significantly lower than that in *T. sinensis*, *D. kuriphilus* galls and galled twigs (Dunn test, *p* < 0.01 for all cases), whereas the α-diversity of the bacterial community in galled twigs was significantly higher than that in *D. kuriphilus*, *T. sinensis* and *D. kuriphilus* galls at the species level (Dunn test, *p* < 0.01 for all cases) (Figure 1). The PCoA indicated that the bacterial community structures of *D. kuriphilus* and *T. sinensis* clearly differed from those of the other groups (Figure 1). These results suggest that *D. kuriphilus* adults, *T. sinensis*, *D. kuriphilus* galls and galled twigs may provide a unique habit for their own bacterial community.

### 3.2. Unique, Common and Predominant Bacterial Species Associated with D. kuriphilus, T. sinensis, D. kuriphilus Galls and Galled Twigs

**Figure 2:**
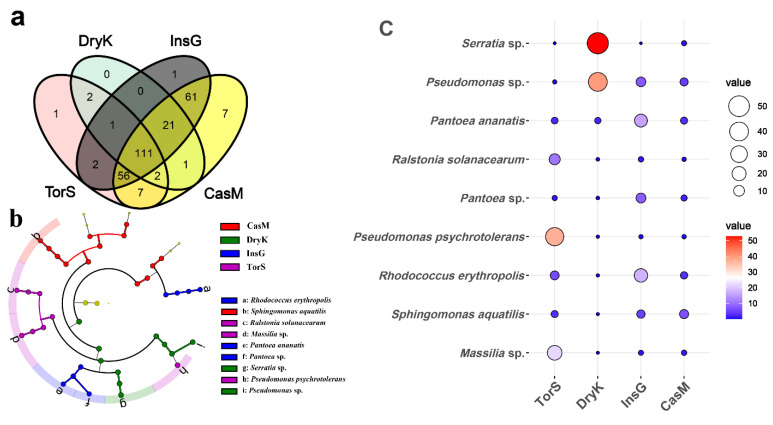
The differences in bacterial community among *Dryocosmus kuriphilus*, *Torymus sinensis*, *D. kuriphilus* galls and the galled twigs of *Castanea mollissima*. (**a**) The number of unique and common bacterial species in *D. kuriphilus*, *T. sinensis*, *D. kuriphilus* galls and galled twigs at the species level. The number shows the number of bacterial species unique or common to *D. kuriphilus*, *T. sinensis*, *D. kuriphilus* galls and galled twigs. (**b**) The LEFSE plot of the predominant bacteria in *D. kuriphilus*, *T. sinensis*, *D. kuriphilus* galls and galled twigs. The cladogram levels, from the inner to outer rings, stand for phylum, class, order, family, genus and species. The green, purple, blue and red nodes of the cladogram show the predominant bacteria in *D. kuriphilus*, *T. sinensis*, *D. kuriphilus* galls and galled twigs from the phylum to species level, respectively. The yellow nodes show the non-dominant bacteria in *D. kuriphilus*, *T. sinensis*, *D. kuriphilus* galls and galled twigs. The letters from a to i show the predominant bacterial species in *D. kuriphilus*, *T. sinensis*, *D. kuriphilus* galls and galled twigs. (**c**) The bubble chart of the relative abundance of the predominant bacteria in *D. kuriphilus*, *T. sinensis*, *D. kuriphilus* galls and galled twigs at the species level. The area and color of the circles show the relative abundance of the predominant bacteria in *D. kuriphilus*, *T. sinensis*, *D. kuriphilus* galls and galled twigs. The relative abundance is expressed as the percentage of predominant bacteria in the total bacteria. DryK, TorS, InsG and CasM represent *D. kuriphilus*, *T. sinensis*, *D. kuriphilus* galls and galled twigs, respectively.

A total of 111 bacteria were common to *D. kuriphilus* adults, *T. sinensis*, *D. kuriphilus* galls and galled twigs at the species level (Figure 2). The numbers of unique bacteria in *D. kuriphilus*, *T. sinensis*, *D. kuriphilus* galls and galled twigs were zero, one, one and seven, respectively (Figure 2). These results imply that the bacteria could be transmitted horizontally among *D. kuriphilus* adults, *T. sinensis*, *D. kuriphilus* galls and galled twigs through trophic relationships.

The LEFSE analysis indicated that a total of two phyla, three classes, seven orders, ten families, eight genera and nine species were predominant in the bacterial communities of *D. kuriphilus*, *T. sinensis*, *D. kuriphilus* galls and galled twigs (Figure 2). The bacterial community of *D. kuriphilus* was dominated by one phylum, one class, two orders, two families, two genera and two species (Figure 2). The bacterial communities of *T. sinensis* and *D. kuriphilus* galls were dominated by one order, two families, two genera and three species, whereas the bacterial community of galled twigs was dominated by one phylum, two classes, three orders, four families, two genera and one species (Figure 2).

For *D. kuriphilus*, the relative abundance of the predominant bacterial species *Serratia* sp. and *Pseudomonas* sp. was 52.71% and 40.1%, respectively (Figure 2 and Table 2). For *T. sinensis*, the relative abundance of the predominant bacterial species *Pseudomonas psychrotolerans*, *Massilia* sp. and *Ralstonia solanacearum* was 37.16%, 21.98% and 10.77%, respectively (Figure 2 and Table 2). For *D. kuriphilus* galls, the relative abundance of the predominant bacterial species *Rhodococcus erythropolis*, *Pantoea ananatis* and *Pantoea* sp. was 17.81%, 15.93% and 8.03%, respectively (Figure 2 and Table 2). For galled twigs, the relative abundance of the predominant bacterial species *Sphingomonas aquatilis* was 6.35% (Figure 2 and Table 2). These predominant bacteria have never been reported in *D. kuriphilus* galls and *T. sinensis* prior to this study (Table 2).

## 4. Discussion

### 4.1. Possibility of Horizontal Transmission of Bacteria among D. kuriphilus, T. sinensis, D. kuriphilus Galls and Galled Twigs

*D. kuriphilus*, *T. sinensis*, *D. kuriphilus* galls and galled twigs shared most bacteria at the species level, thus suggesting that the bacteria might be transmitted horizontally among *D. kuriphilus* adults, *T. sinensis* adults, *D. kuriphilus* galls and galled twigs. We speculated that ecological interactions might be associated with the potential horizontal transmission among *D. kuriphilus*, *T. sinensis*, *D. kuriphilus* galls and galled twigs.

The water and most of the nutrients of *D. kuriphilus* galls are obtained from the host plants via the vascular system [8], and the transportation of these substances may provide favorable conditions for the horizontal transmission of bacteria. For example, previous studies have confirmed that the species of the *Serratia* and *Pseudomonas* genera can migrate from root to leaf via the transportation of substances through the vascular system, according to green fluorescent protein labeling and β-glucuronidase staining [36,37]. Furthermore, for the *D. kuriphilus* adults, there is a subset of bacteria from *D. kuriphilus* galls. *D. kuriphilus* galls are the sole food source for *D. kuriphilus*, and plant endophytes can enter the digestive system of phytophagous insects through feeding [38,39,40]. Thus, the feeding relationship between *D. kuriphilus* and *D. kuriphilus* galls may be beneficial for the horizontal transmission of bacteria, and most bacteria of *D. kuriphilus* may be derived from *D. kuriphilus* galls. *T. sinensis* is an important natural enemy of *D. kuriphilus*, and feeds on the *D. kuriphilus* larvae. Our studies suggest the horizontal transmission of *Wolbachia* between the gall wasp *Andricus mukaigawae* and parasitoids *Torymus* sp. [40]. Previous studies have confirmed the horizontal transmission of bacteria between insect hosts and three parasitoids using florescence in situ hybridization and transmission electron microscopy [41]. We suggest that the parasitic relationship between *T. sinensis* and *D. kuriphilus* may provide a potential route for the horizontal transmission of bacteria. Furthermore, some species of *Torymus* can feed on plant material [12] and *T. sinensis* may acquire the bacteria from overwintering withered galls.

### 4.2. Differences in Bacterial Community Structures among D. kuriphilus, T. sinensis, D. kuriphilus Galls and Galled Twigs

The chemical characteristics of *D. kuriphilus* galls may be associated with the differences in bacterial community structures between *D. kuriphilus* galls and galled twigs. First, the concentrations of auxins and cytokinins in galled twigs are different from those in galls, such as midge [42], aphid [43], fly [44], psyllid [45,46], leafhopper [47], sawfly [48], *L. invasa* [49] and *D. kuriphilus* galls [50]. The auxins and cytokinins can directly or indirectly affect the physiology of bacteria. For example, the auxins and cytokinins can directly affect the stress-related genes of bacteria as important signaling molecules [51]. Moreover, auxins and cytokinins can indirectly affect bacteria through communicating with jasmonic acid and salicylic acid signaling pathways, which are two major pathways involved in the plant’s defense against attacks by pathogens [52,53]. Second, the concentrations of amino acids, carbohydrates, lipids, lignin and secondary metabolites in cynipid galls markedly differ from those in ungalled host plant tissue [8,54,55,56,57,58]. The nutrients in cynipid galls provide unique carbon and nitrogen sources for the bacterial community, and high levels of tannin, polyphenol oxidase and reactive oxygen species in cynipid galls can inhibit the growth of some bacteria [54,59,60]. Thus, we suggest that the compositions and concentrations of chemical substances in *D. kuriphilus* galls provide a special habitat for the bacteria associated with *D. kuriphilus* galls.

The differences in bacterial community composition between *D. kuriphilus* and *T. sinensis* may be associated with the life history of *D. kuriphilus* and *T. sinensis*. First, *D. kuriphilus* is an herbivorous insect and *T. sinensis* is a parasitoid [61]. The differences in food sources may affect the differences in bacterial community composition between *D. kuriphilus* and *T. sinensis*. Second, the species barriers between *D. kuriphilus* and *T. sinensis* may prevent the colonization of bacteria [12,62]. Third, *T. sinensis* adults emerge from the withered overwinter galls whereas *D. kuriphilus* adults emerge from newly formed galls [9]. The overwintering withered galls were induced by *D. kuriphilus* in the previous year, and the environment of *D. kuriphilus* is different from that of *T. sinensis*. Thus, we suggest that the food sources, species barriers and gall environment may affect the differences in bacterial communities between *D. kuriphilus* and *T. sinensis*.

### 4.3. Predominant Bacteria Associated with D. kuriphilus

The *Serratia* sp. and *Pseudomonas* sp. are predominant in *D. kuriphilus* and widely distributed in galling insects, including *D. kuriphilus*, gall midges, gall aphids, gall sawflies and thrips [17,18,23,24,25,26,27,63,64]. Many species of the *Serratia* and *Pseudomonas* genera are plant pathogenic bacteria [65]. *D. kuriphilus* lay eggs in the buds of host plants through its ovipositor, and the buds come into contact with the ovipositor and ovipositional fluid [66]. Furthermore, *D. kuriphilus* galls come into contact with the feces and saliva of *D. kuriphilus* [67]. We speculate that the *Serratia* sp. and *Pseudomonas* sp. of *D. kuriphilus* may be plant pathogenic bacteria, and *D. kuriphilus* adults may be potential vectors of these plant pathogens.

Moreover, some species of the *Pseudomonas* genus can synthetize auxins and cytokinins, both of which have been identified in various galling insects, including gall midges [42], gall aphids [43], fruit flies [44], leafhoppers [47], gall sawflies [48] and psyllids [45,46]. Recent studies have suggested that the fast growth of gall induction may not be consistently mediated by a bacterial symbiont [27]. However, it remains unclear whether the bacteria are associated with the auxins and cytokinins in galling insects. In future studies, we plan to culture the *Pseudomonas* sp. in *D. kuriphilus* and explore its potential roles.

## 5. Conclusions

In conclusion, we provide the first evidence that *D. kuriphilus* shares most bacterial species with *T. sinensis*, *D. kuriphilus* galls and galled twigs, and are dominated by *Serratia* sp. and *Pseudomonas* sp. Furthermore, the bacterial community structures of *D. kuriphilus* and *T. sinensis* clearly differ from those of the other groups.

We suggest that vascular transportation, feeding and parasitic relationships may be associated with horizontal transmission among *D. kuriphilus*, *T. sinensis*, *D. kuriphilus* galls and galled twigs. Furthermore, the chemical characteristics of *D. kuriphilus* galls may be associated with the differences in bacterial community structures between *D. kuriphilus* galls and galled twigs. The food sources, species barriers and gall environment may affect the differences in bacterial communities between *D. kuriphilus* and *T. sinensis*. In addition, we suggest that *D. kuriphilus* may be a potential vector of plant pathogens.

## Figures and Tables

**Table 1:** The total number of bacteria in *Dryocosmus kuriphilus*, *Torymus sinensis*, *D. kuriphilus* galls and the galled twigs of *Castanea mollissima* from phylum to OTU levels.

	Phylum	Class	Order	Family	Genus	Species	OTU
*T. sinensis*	10	15	54	80	125	182	221
*D. kuriphilus*	8	12	40	64	98	138	162
*D. kuriphilus* galls	13	19	60	96	170	253	350
Galled twigs	14	20	61	100	176	266	366
Total	14	20	63	103	181	273	373

**Table 2:** A report about the genus of the predominant bacteria in *Dryocosmus kuriphilus*, *Torymus sinensis*, *D. kuriphilus* galls and the galled twigs of *Castanea mollissima*.

Genus of Predominant Bacteria	Reported in *T. sinensis*	Reported in Galling Insects	Reported in *D. kuriphilus galls*	Reported in *C. mollissima*
TorS group				
*Massilia*	No	*Leptocybe invasa* (Liu et al., 2021)	No	No
*Ralstonia*	No		No	No
*Pseudomonas*	No	*D. kuriphilus* (Iskender et al., 2017), *L. invasa* (Liu et al., 2021), Gall midges (Bansal et al., 2014; Ojha et al., 2017), Gall aphids (Medina et al., 2011; Wu et al., 2018), Galling sawflies (Michell and Nyman, 2021), Thrips (Hammer et al., 2021)	No	No
Dryk group				
*Serratia*	No	Gall wasps (Liu et al., 2021), Gall aphids (Amit et al., 2017; Medina et al., 2011; Wu et al., 2018), psyllids (Morrow et al., 2017)	No	Chen et al., 2019
*Pseudomonas*	No	*D. kuriphilus* (Iskender et al., 2017), *L. invasa* (Liu et al., 2021), Gall midges (Bansal et al., 2014; Ojha et al., 2017), Gall aphids (Medina et al., 2011; Wu et al., 2018), Galling sawflies (Michell and Nyman, 2021), Thrips (Hammer et al., 2021)	No	Ni, 1998;
InsG group			No	
*Pantoea*	No	*L. invasa* (Liu et al., 2021), Gall midges (Bansal et al., 2014; Ojha et al., 2017), Thrips (Hammer et al., 2021)	No	Zhang et al., 2019
*Rhodococcus*	No	Gall aphids (Wu et al., 2018)	No	No
CasM group				
*Sphingomonas*	No	*L. invasa* (Guo et al., 2020), Galling sawflies (Michell and Nyman, 2021), Gall aphids (Wu et al., 2018)	No	Chen et al., 2019

## Data Availability

The raw data were deposited into the NCBI Sequence Read Archive (SRA) database under BioProject accession number PRJNA780913.

## Supplementary Materials

The following are available online at https://www.mdpi.com/article/10.3390/insects13010086/s1: Figure S1, the galls induced by *Dryocosmus kuriphilus* and galled twigs.
