# Supplementary figures and images for: The Diversity of Bacteria Associated with the Invasive Gall Wasp Dryocosmus kuriphilus, Its Galls and a Specialist Parasitoid on Chestnuts
Source: Insects. 2022 Jan 13;13(1):86. doi: 10.3390/insects13010086 (PMC8778799; doi:10.3390/insects13010086)

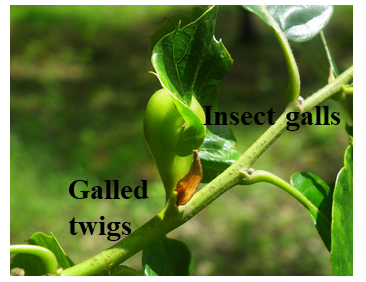

Supplement: Supplementary file 1 [file insects-13-00086-s001.zip › insects-sup-1511563/Figure S1 The galls induced by Dryocosmus kuriphilus and galled twigs.tif]
